# Effects of terrorism-related attacks and family planning interventions on the uptake of modern contraceptives in Burkina Faso: a nationwide interrupted time-series analysis (2013–2023)

**DOI:** 10.1136/bmjopen-2026-116769

**Published:** 2026-08-31

**Authors:** Cheick Tiendrebeogo, Federica Fregonese, Neda Firouraghi, Thomas Druetz

**Affiliations:** 1 Social and Preventive Medicine, University of Montreal School of Public Health, Montreal, Quebec, Canada; 2 University of Montreal School of Public Health, Montreal, Quebec, Canada; 3 London School of Hygiene & Tropical Medicine, London, UK; 4 Tulane University School of Public Health and Tropical Medicine, New Orleans, Louisiana, US

**Keywords:** Health Services Accessibility, Health policy, International health services

## Abstract

**Abstract:**

**Objective:**

To investigate the effect of terrorism-related attacks on modern contraceptive uptake and to assess the effects of recent family planning (FP) interventions in Burkina Faso.

**Design:**

Nationwide longitudinal study using a multiple interrupted time-series analysis.

**Setting:**

Public primary healthcare centres and district hospitals in Burkina Faso, from January 2013 to December 2023.

**Data sources:**

Monthly data on new FP users were drawn from Burkina Faso’s National Health Information System, covering 132 months. Data on terrorist attacks were extracted from the Armed Conflict Location and Event Data Project.

**Exposures:**

The main exposure was the occurrence of terrorist attacks at the commune-month level. Secondary exposures were the 2020 FP user-fee exemption policy and national family planning weeks (NFPWs).

**Primary and secondary outcome measures:**

The primary outcome was the monthly count of new FP users used as an indicator of modern contraceptive uptake. Secondary measures were predicted gains and losses in visits by new FP users associated with terrorist attacks, user-fee removal and NFPWs.

**Results:**

The monthly number of visits by new FP users decreased by 13% (95% CI 11% to 15%) in the month of and following a terrorist attack. NFPWs were associated with a 152% increase in new users (95% CI 147% to 156%), while the 2020 FP fee exemption policy was associated with a 22% immediate increase (95% CI 18% to 25%). Predicted gains were greater for NFPWs (+4 31 846 new users) than for the free FP policy (+3 52 006). Terrorist attacks were associated with a predicted loss of 133 688 new users since January 2013.

**Conclusion:**

Terrorist attacks were associated with reduced modern contraceptive uptake in Burkina Faso, likely through disruptions in both service supply and access. Combining user-fee removal with active outreach strategies such as NFPWs may help sustain reproductive health service coverage in settings affected by protracted insecurity.

**STRENGTHS AND LIMITATIONS OF THIS STUDY**

- The multiple interrupted time-series design with a negative binomial model, commune-level fixed effects, calendar-month seasonality terms, a first-order autocorrelation parameter and cluster-robust variance estimators allows robust control of unobserved time-invariant heterogeneity, seasonality and within-commune serial dependence.
- Linking National Health Information System and Armed Conflict Location and Event Data data at the commune-month level enabled measurement of exposure to terrorist attacks and fatalities, while a supplementary analysis assessed potential reporting bias in insecure areas.
- The primary outcome (monthly visits by new FP users) captures contraceptive uptake but does not measure continuation, method switching or couple-years of protection and is triangulated descriptively with the total volume of FP service contacts in online supplemental appendix 4.
- The ecological design precludes inference at the individual level, routine service data may be subject to under-reporting in conflict-affected areas and facility-level random effects could not be modelled because facility identifiers are not retained in the available commune-month dataset.

## Introduction

 In 2015, the WHO estimated that only 29% of women of reproductive age (aged 15–49 years) in Africa were using a contraceptive method.^1^ This limited coverage contributes to persistently high fertility rates, particularly in sub-Saharan Africa (SSA), where the total fertility rate stands at 5.4 children per woman^2^; however, these high fertility levels do not always reflect women’s reproductive intentions.^3^ Many report having had more children than desired, and approximately 24% of married women in the region have an unmet need for contraception (UNC): They want to stop or delay childbearing but are not using any method of contraception.^3 4^ This discrepancy constitutes a violation of women’s fundamental rights to bodily autonomy, particularly their right to decide the number and timing of their children.^4 5^

Studies suggest that restricted access to family planning (FP) increases the risk of unintended pregnancies, which can lead to closely spaced births, unsafe abortions and poorer maternal health outcomes.^6–9^ In low- and middle-income countries, UNC contributes to an estimated 299 000 maternal deaths annually,^10 11^ and in SSA, it is the leading cause of unsafe abortions.

Barriers to accessing contraceptive methods are well documented^12 13^ and include religious and cultural beliefs,^14 15^ resistance from male partners,^16 17^ cultural pressure for women to have large families^18^ and widespread misconceptions about side effects.^19 20^ Armed conflicts and insecurity can further compound these barriers,^16 21 22^ even as crises and conflicts have a negative impact on sexual and reproductive health and rights (SRHR) services^8^ and childbearing intentions.^7 23–27^

In 2024, only 28% of women in sexual relationships with men were using a modern contraceptive method,^28^ and nearly 20% had a UNC.^29^ Distance to health facilities, limited decision-making autonomy, cultural beliefs, lack of partner support and inadequate training of health workers in contraceptive logistics management all contribute to this unmet need.^13 30 31^

Over the past 10 years, Burkina Faso has faced a surge in terrorist attacks and criminal activities of low-to-moderate intensity in an attempt to destabilise the state. These attacks led to the displacement of over two million people^32 33^ and plunged much of its territory into a state of insecurity.^34 35^ This instability affected the healthcare system in general and FP services in particular,^36 37^ including destruction of health infrastructure, flight/displacement of healthcare personnel,^38–40^ near-total closure of health facilities in some areas^41^ and disruption of FP service delivery.^42 43^

As highlighted in Kruk’s conceptual framework, these physical, psychological, socio-cultural and logistical barriers further undermine health system resilience^44^ and threaten to reverse recent progress in access to SRHR.^45–48^ Since July 2020, SRHR services, including FP, have been provided free of charge nationwide in public health facilities as part of the country’s efforts towards universal health coverage.^48 49^ Since 2012, national family planning weeks (NFPWs) have been held regularly to raise awareness and support of FP services and provide free contraceptives.^50^ NFPWs are targeted toward women who are particularly likely to have a UNC, like young women and those who live in rural areas.^51 52^ These initiatives have been associated with increased use of contraceptives and a reduction of UNC in Burkina Faso and elsewhere.^53–56^

Although terrorist attacks are likely to affect contraceptive use, empirical evidence is limited. While the effects of user-fee exemptions on maternal and child health services are well documented, and recent studies^57–59^ have shown how the Sahelian security crisis has reshaped health sector priorities and financing, these dynamics have largely been examined in isolation. This study aims to fill this gap in the literature by measuring the extent to which terrorist attacks have eroded the gains in contraceptive uptake triggered by free FP policies and NFPWs. Methodologically, it is the first—to our knowledge—to conduct a multiple interrupted time series analysis to examine the association between violent attacks and contraceptive use in Burkina Faso.

The primary objective is to quantify the effect of terrorism-related insecurity on the rate of visits for contraceptive use in the public health facilities of Burkina Faso, analysing the magnitude and variation of this impact across the country. The secondary objective is to measure the presumed gains in contraceptive use due to the FP service exemption policy and NFPWs.

## Methodology

### Study design

This is a longitudinal study using multiple interrupted time series performed on routine administrative data collected monthly in all public health centres in Burkina Faso. Health services data from January 2013 to December 2023 (132 months) were collated at the commune level (the smallest administrative unit in the country). This study has followed the REporting of studies Conducted using Observational Routinely-collected Data (RECORD) guidelines.

### Data sources

Data on visits of contraceptive users were obtained from Burkina Faso’s National Health Information System (NHIS) database. These consultation data are collected monthly at all public health centres in Burkina Faso: ~2300 primary healthcare facilities, 49 district-level medical centres, 9 regional hospitals and 6 university hospitals. Each month, data are sent to the management staff of health districts, where they are compiled and checked for completeness. Next, they are sent to the central level, which ensures that they are horizontally and vertically integrated in the main dataset. Regular audits are carried out by the Ministry of Health to ensure quality and reliability. Other studies have confirmed the validity of these data for measuring trends in consultation rates.^47 60 61^ Previous studies that used routine NHIS data to assess the impact of interventions on the utilisation of health services have reported high levels of completeness and temporal consistency of monthly facility reports in Burkina Faso.^57 62^ Additional work on the performance of the NHIS and spatiotemporal modelling of malaria incidence also concludes that, despite known limitations, routine data are sufficiently complete and consistent to support trend analysis.^63 64^ The NHIS platform presents data obtained from health registers, aggregated as monthly counts at the facility level.

Data on exposure to terrorist attacks were sourced from the *Armed Conflict Location and Event Data* (ACLED) project, which was established in 2005 to track global violent events of a political or terrorist nature.^65^ ACLED gathers detailed information on the date, location, actors, violence type and victim count for each event.^66 67^ It covers a wide range of political actors, including governments, rebel groups and civilians, drawing data from diverse sources such as news reports, non-governmental organisation publications and security alerts.^66^ The project documents nine types of conflict events, grouped into four broader categories: battles, remote violence, violence against civilians and ‘other’. For analysis purposes, riots, protests and certain sub-events such as ‘agreement’, ‘change to group/activity’, ‘government regains territory’, ‘non-state actor overtakes territory’, ‘nonviolent transfer of territory’ and ‘other’ were excluded. ACLED is a widely used open-source database, valued for its credibility, robustness and scholarly relevance, overcoming potential biases inherent in secondary data collection.^33 68–72^

Throughout this study, the unit of analysis is the commune-month—that is, one observation per commune (the smallest administrative subdivision of Burkina Faso; n=351) for each calendar month over 132 months. Each commune-month contains the count of visits by new FP users aggregated across all public health facilities located in that commune.

The ACLED and NHIS databases (both cover the same analysis period, from January 2013 to December 2023) were merged at the commune-month level using the date and geo-referenced information (longitude and latitude) of the attacks to allocate them to a commune-month in the NHIS database.

The matching variables (13 regions and 351 communes) were carefully checked for correspondence between the ACLED and NHIS datasets. The spatial delimitation of the 351 communes was obtained from the *Humanitarian Data Exchange* platform, hosted by the United Nations Office for the Coordination of Humanitarian Affairs, triangulated with that of the WHO and of the Burkina Faso Demographic and Health Information System. The data linkage process and analytical sample selection are illustrated in online supplemental appendix figure 1.

The dates of the NFPWs were compiled from the websites of the Ministry of Health of Burkina Faso and the United Nations Population Fund.^50 73^ Each commune’s population was obtained from the 2019 General Population and Housing Census of Burkina Faso.^74^

### Outcome and exposure variables

The NHIS defines a ‘new user’ as any woman recorded for the first time as adopting a modern contraceptive method at a given health facility. The primary outcome was the monthly number of visits for new FP users per commune-month, used as an indicator of modern contraceptive uptake or adoption. Because this indicator does not capture continuation or follow-up visits, online supplemental appendix 4 descriptively reports the combined annual volume of visits for new and old/continuing FP users (all FP users) to contextualise the broader volume of FP service contacts recorded in routine data.

The main exposure variable was the occurrence of a terrorist attack, defined as an act of violence involving a jihadist group or a group of unidentified armed individuals, regardless of whom they were perpetrated against (civilians, armed forces, self-defence militias, etc.). It was defined as a binary categorical variable taking the value ‘1’ if the commune suffered at least one terrorist attack during that month or the preceding month and ‘0’ otherwise. This temporal window is consistent with most empirical studies conducted in SSA or in Syria, precisely to capture short-term variations in maternal health indicators.^22 75–77^

Two secondary exposures were considered. First, we considered all the communes exposed to NFPWs during active nationwide campaigns to raise awareness and distribute free contraceptive methods. Between January 2013 and December 2023, 12 campaigns were conducted, each lasting 1–2 weeks. NFPW exposure was expressed as a binary categorical variable: ‘1’ in NFPW campaign months and ‘0’ otherwise. Second, the introduction of free FP services in July 2019 or July 2020 (depending on the region) was considered and expressed with two variables: 1) a binary categorical variable that took the value ‘0’ before FP services were free and ‘1’ when no-cost visits were implemented and 2) a continuous variable (expressed as months after free FP starting) measuring no-cost FP trends incrementally.

### Analysis

Interrupted time series were fitted using a longitudinal panel regression framework with commune and month fixed effects within a negative binomial specification to address overdispersion. The analysis was conducted at the commune-month level to assess changes in FP service use at the population level, regardless of the number or type of health facilities operating within each commune. A first-order autocorrelation parameter and cluster-robust variance estimators were used to account for residual within-commune correlation over time, while commune and month fixed effects controlled for unobserved commune-level heterogeneity and seasonality. A baseline trend parameter was also included in the model.

The model also incorporates two dummy variables to assess (i) the immediate effect of introducing the free FP policy and (ii) the change in the baseline trend after its introduction. Finally, a dummy variable was added to measure the average treatment effect of being exposed to NFPWs. Quadratic terms were tested to allow a curvilinear function of the baseline and post-gratuity trends. Residual serial correlation was reduced by using fixed effects and adding a first-order autocorrelation parameter. Coefficients were expressed as incidence rate ratios. The models derive from:


$$\begin{array}{l} Y_{t}=\beta_{0}+\beta_{1}.T+\beta_{2}.T^{\left[sq\right]}+\beta_{3}.D_{t}+\beta_{4}.G_{t}+\beta_{5}.(T\text{-}T_{j})+\\ \beta_{6}.(T\text{-}T_{j}{)}^{\left[sq\right]}+\beta_{7}.Q_{t}+\beta_{8}.S+\beta 9.Y_{t-1}+\in_{t} \end{array}$$

where (Y_t_) represents the dependent variable, (T) the time ranging from 1 to 132, (D_t_) the exposure to the free FP policy, (T_j_) the time when the free FP policy was introduced, (G_t_) the exposure to NFPW, (Q_t_) the effect of seasonality, (S) the fixed commune level and (ϵ_t_) corresponds to the random error.

Based on the model, we measured the national, longitudinal population effect of exposure to terrorist attacks, user fee removal policy and NFPWs. Using Stata’s margins and nlcom commands, the crude total gains and losses in the volume of new FP users were predicted using the actual dates of the events under study.

Sensitivity analyses were performed to test the hypothesis that attacks can lead to the temporary closure of health centres, causing a reduction in the rate of FP consultations. A regression model was fitted using a Poisson distribution with the dependent variable being the number of health facilities per commune that transmitted consultation data. Fixed effects were included at the commune level.

All analyses were performed using Stata software, and maps were generated using QGIS software. The threshold of statistical significance was set at 5% (bilateral).

### Patient and public involvement statement

Patients and members of the public were not used in the design, conduct, reporting or dissemination of this research. Use of healthcare services by patients was routinely collected by healthcare providers and analysed; however, data were aggregated, and individual patients cannot be identified from the reported data.

### Reflexivity statement

The research team has strong ties to Burkina Faso. The first author (CT), a Burkinabe physician, brings in-depth knowledge of the health system and local context, while the senior author (TD) has conducted collaborative health systems research in the country for over a decade. The Ministry of Health provided access to NHIS data through a formal agreement and reviewed the operational definitions of study indicators. This contextual expertise informed the study design, interpretation of findings and manuscript preparation.

## Results

### Insecurity crisis in Burkina Faso

**Figure 1:**
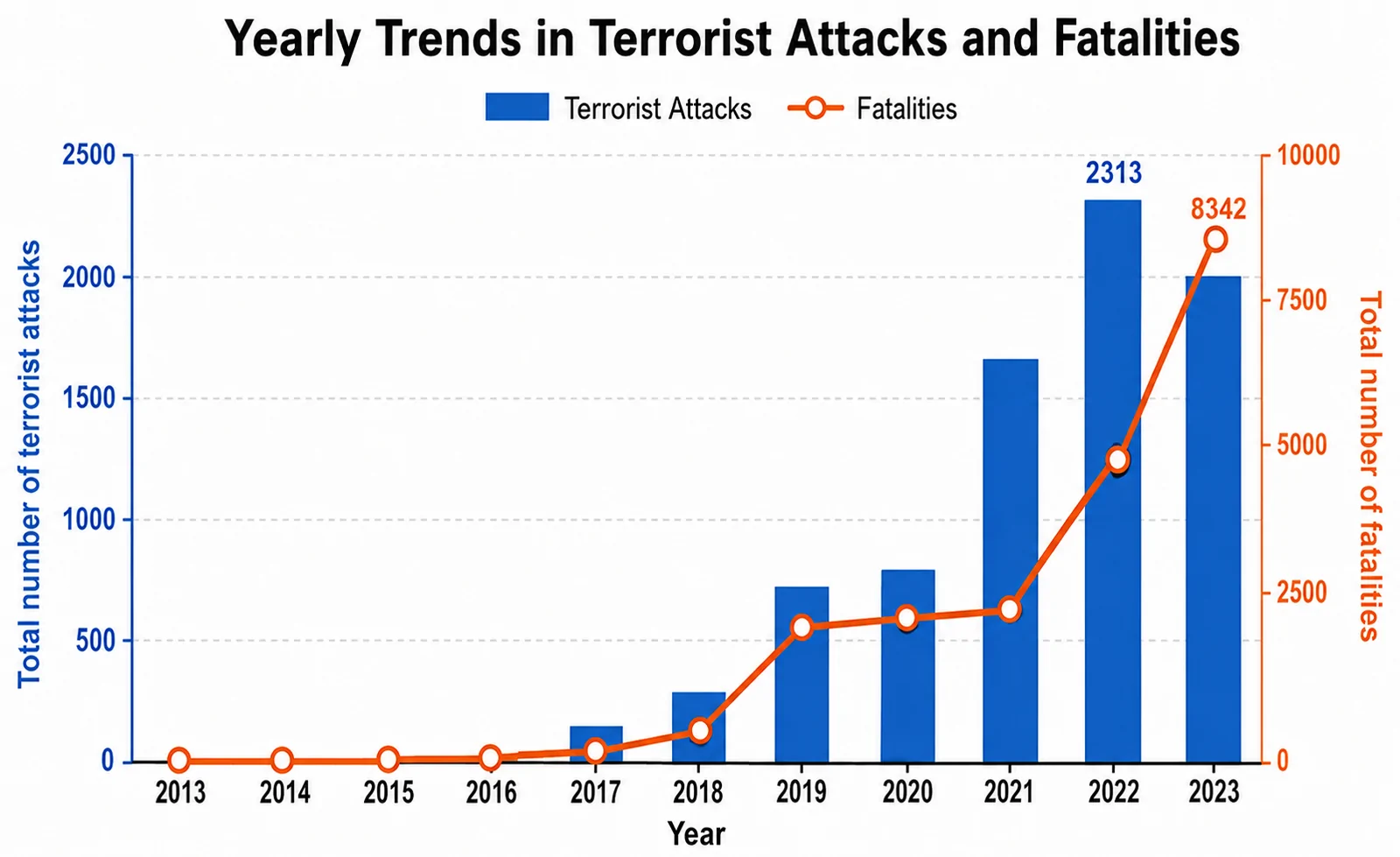
Yearly number of terrorist attacks and civilian fatalities in Burkina Faso, per year, 2013–2023. Data source: ACLED. ACLED, Armed Conflict Location and Event Data.

**Figure 2:**
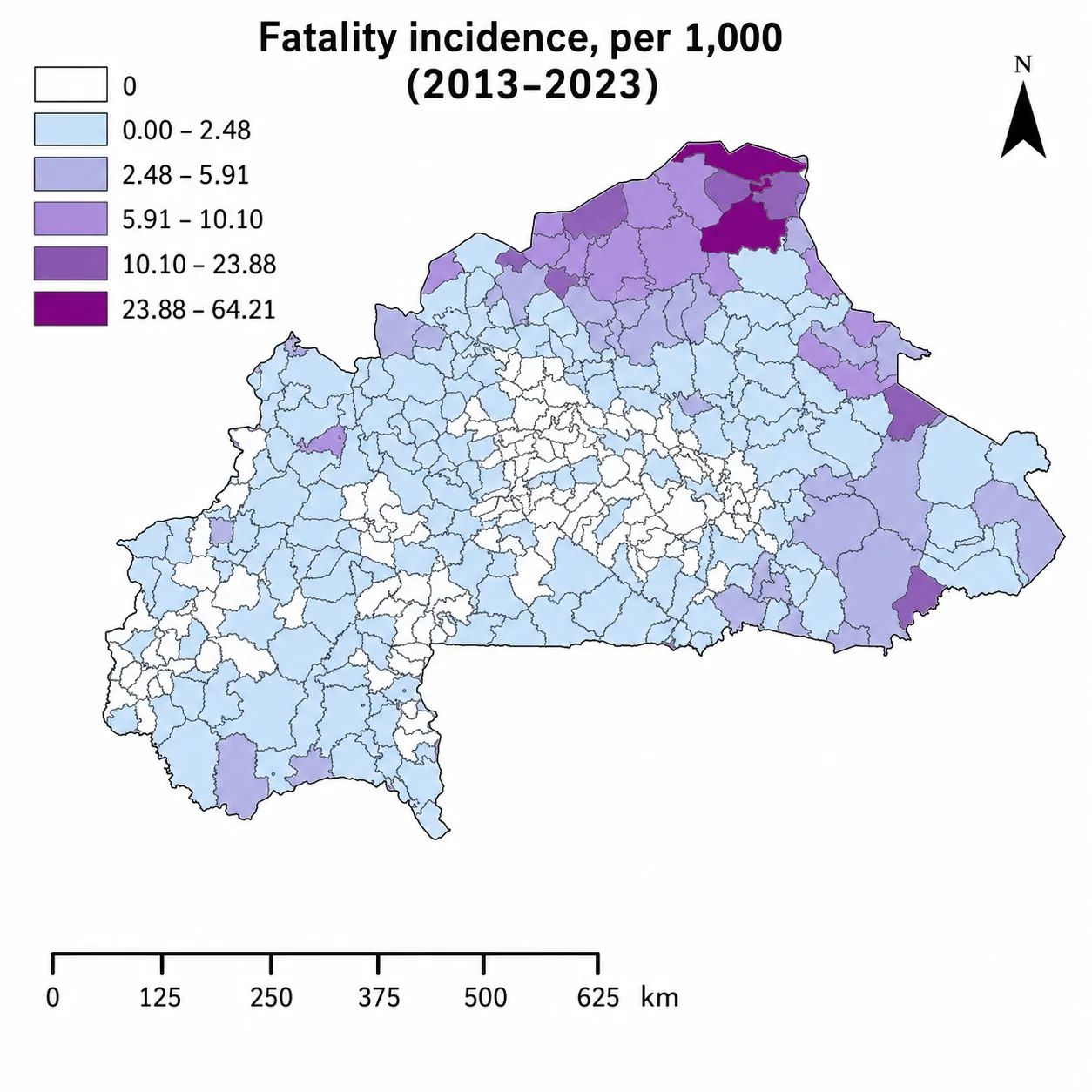
Map of Burkina Faso with cumulative density of fatalities due to terrorist attacks per commune, 2013–2023. Map produced by the authors using QGIS. Commune-level boundaries were obtained from the Humanitarian data exchange program (HDX) from the United Nations Office for the Coordination of Humanitarian Affairs, and fatality counts were derived from ACLED data. No third-party copyrighted basemap is displayed. Data source for fatalities: ACLED. ACLED, Armed Conflict Location and Event Data.

In 2013–2023, terrorist attacks increased from 5 in 2013 to over 2000 in 2023 (figure 1). From 2017 onwards, there was a steep increase in attacks, from 25 in 2016 to 103 in 2017. This 300% increase marks a breaking point in the trend observed in previous years. The number of civilian victims has been following a similar trend, with a gradual increase and a marked acceleration from 2019 onwards, along with a territorial expansion of unsafe areas in Burkina Faso (figure 2).

Terrorist attacks were recorded in 117 (89%) of the 132 observation periods. A total of 254 (72%) of the 351 communes recorded at least one attack. In these exposed communes, the average number of attacks per commune per month was 0.17, but the SD of 0.84 indicates a high level of variability, with some communes having experienced more attacks than others.

### Effects of terrorist attacks on contraceptive use

The average effects of terrorist attacks on contraceptive use are shown in table 1. The monthly number of visits by new FP users in a commune decreased by 13% (95% CI 11% to 15%) during the months when a terrorist attack was recorded. The sensitivity analysis shows that attacks were also associated with a reduction in the number of health facilities reporting data (−15%, 95% CI 14% to 17%) (online supplemental appendix 2).

**Table 1:** Effects of attacks and interventions on new FP users in Burkina Faso, 2013–2023

	IRR	95% CI
Secular trend	0.989^*^	0.989 – 0.990
Secular trend (quadratic)	1.000^*^	1.000 – 1.000
Post-gratuity trend	1.007^*^	1.005 – 1.009
Post-gratuity trend (quadratic)	0.999^*^	0.999 – 0.999
Terrorist attacks	0.872^*^	0.856 – 0.889
NFPW	2.521^*^	2.467 – 2.563
Free FP	1.215^*^	1.183 – 1.247

The model was fitted with a negative binomial distribution, robust variance estimators and fixed effects at the commune level; it was also adjusted for seasonal variations (months). Unit of analysis is the commune-month.

* p<0.001.

CI, confidence interval; FP, family planning; IRR, incidence rate ratio; N, number; NFPWs, national family planning weeks.

### Effects of gratuity initiatives on contraceptive use

**Figure 3:**
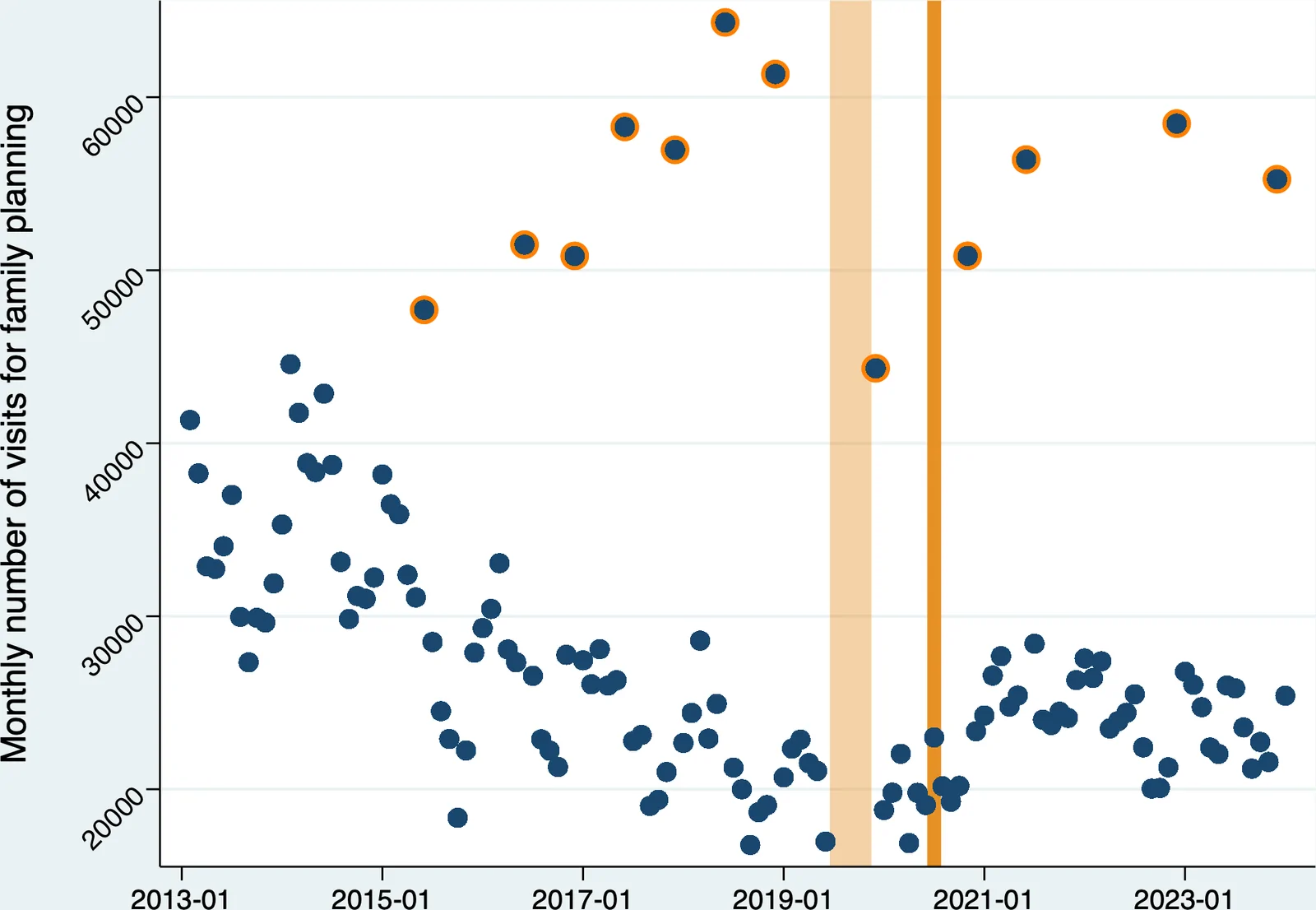
Crude total number of new FP users in Burkina Faso, 2013–2023, by month. The blue dots are observations under normal conditions, while the orange dots are observations in months during which occurred national family planning weeks (NFPWs). The time period in light orange corresponds to the partial introduction of free healthcare (pilot project), which coincided with a national strike in health facilities, resulting in missing data for 4–5 months. The time period in dark orange represents the national introduction of free family planning services. FP, family planning.

Between January 2013 and December 2023, Burkina Faso held NFPWs on 12 occasions, always in May, June, November or December. A visual examination of the crude trend in the total number of new FP users in Burkina Faso suggests a marked increase in each month during which an NFPW was held (figure 3). The negative binomial regression model indicates that NFPWs increase new FP users by 152% (95% CI 147% to 156%) (table 1). This immediate increase is not statistically different before versus after the introduction of the free FP policy (online supplemental appendix 3).

The introduction of free FP services in July 2020 was accompanied by a more modest immediate increase (jump effect) in the monthly number of visits by new FP users (+22%, 95% CI 18% to 25%). Since this intervention has been sustained since its introduction, it is also possible to examine the repercussions on the trend. Analysis suggests that the introduction of the policy was significantly associated with a slowdown in the downward secular trend. Indeed, while the reduction reached −1.1% per month between 2013 and 2020, this decline has been slowed by ~0.7% each month since July 2020 (table 1).

If we consider total FP user visits rather than new user visits, the trend is no longer systematically downward year after year. The total number of FP visits has fluctuated between 967 000 and 1 166 000 over the years, with the largest volumes recorded in 2014 and 2016 (online supplemental appendix 4).

### Predicted gains and losses in the number of new FP users

**Figure 4:**
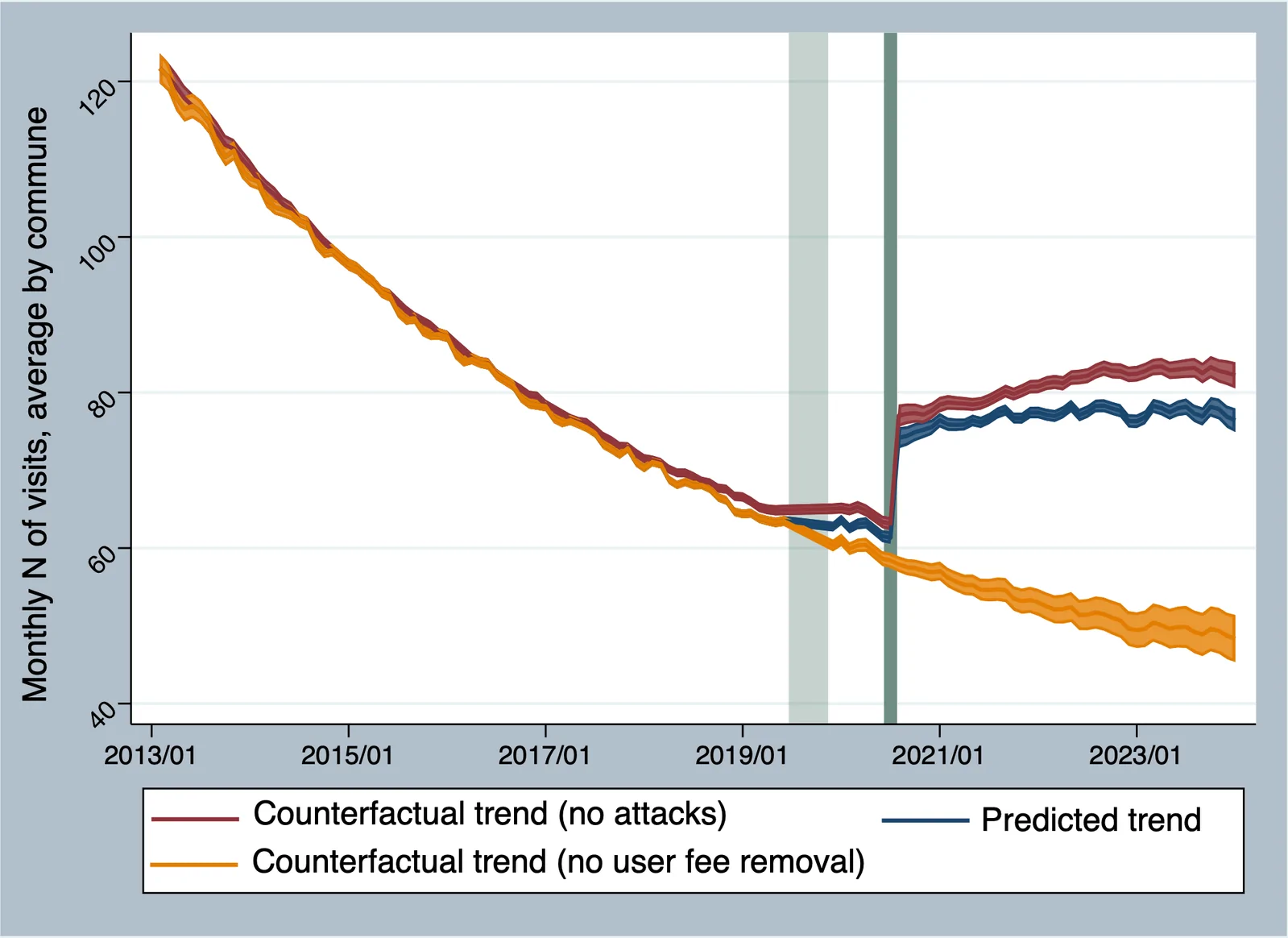
Predicted and counterfactual monthly trends of new FP users per commune-month in Burkina Faso (2013–2023), with 95% CIs, according to exposure to attacks and free FP policy. The predictions are based on a negative binomial model with fixed effects at the commune level, adjusted for seasonal variations and the effects of national family planning weeks (NFPWs). The area in light green represents the partial introduction of free healthcare (pilot project), which coincided with a national strike in health facilities, leading to missing data for 4–5 months. The area in dark green represents the national introduction of free family planning (FP) services. N, Number.

Figure 4 shows the predicted trend of new FP users (per commune-month) based on the actual exposure to terrorist attacks and free FP policy. It also shows two counterfactual trends, without their respective exposure: A visual examination reveals that the volume of new FP users would have been higher in the absence of terrorist attacks, especially since 2018–2019, when the number of attacks surged. It also reveals that the same volume would have been considerably reduced had it not been for the free policy, which reversed the gradual downward trend observed between 2013 and 2020.

The predicted gains and losses in the total number of new FP users attributable to each of the three exposures are shown in table 2. The predicted gains are, in absolute terms, greater for the 12 one-off NFPWs (+ 4 31 846 new users) than for the free policy introduced in 2020 (+ 3 52 006). Exposure to terrorist attacks is associated with an absolute loss of 133 688 new users since January 2013.

**Table 2:** Predicted gains and losses in the total number of new FP users in Burkina Faso, 2013–2023

Exposure	Predicted N of new FP users	95 % CI
NFPWs	431 846	420 040 to 443 652
Free FP policy	352 006	309 134 to 394 877
Terrorist attacks	−133,688	−159 502 to −107 873

CI, confidence interval; FP, family planning;N, number; NFPWs, national family planning weeks.

## Discussion

This study sheds further light on the isolated impact of the security crisis and health policies on the adoption of contraceptive methods by new users. Our results show that the uptake of modern contraception by new users is negatively affected by the occurrence of terrorist attacks and positively affected by initiatives that promoted FP, namely the abolition of direct payment (passive intervention) or national FP weeks (active intervention). These findings should be interpreted in light of the outcome measure used. While new FP users capture contraceptive uptake, they do not fully reflect the overall volume of FP services delivered, which could also be assessed using indicators such as couple-years of protection.

### Terrorist attacks, insecurity and use of FP services

The observed decline in FP use is consistent with the hypothesis of a dual mechanism: a reduction in service supply and a strengthening of access barriers. The effects of these two dynamics are exacerbated in contexts where the demand for FP tends to increase.

First, terrorist attacks and the resulting insecurity are likely to reduce the supply of services and the availability of contraceptives in health facilities. In 2022, it was reported that approximately 500 health centres across the country were either closed or operating at minimal capacity.^41^ Similar situations have been documented in other conflict zones in Africa and the Middle East.^25 78 79^ Health personnel are sometimes targeted in these attacks and may decide to leave the area.^80^ Departure of qualified personnel exacerbates human resource shortages and undermines service delivery, which is also observed in Nigeria, Burundi, Ethiopia and the DRC.^6 75 81–83^ A similar pattern has been reported in Mali, where programme reports and policy briefs indicate that conflict and displacement constrain the availability of SRHR services, including contraception.^84 85^ Insecurity can disrupt supply chains due to the destruction of transport infrastructure and unsafe roads. These disruptions cause stock-outs and further reduce service availability, as documented in Uganda, South Sudan and Mali.^27 39 42^ Our sensitivity analyses showed a 15% reduction in data transmission immediately after a terrorist attack, supporting the hypothesis that the observed reduction in the number of new FP users in Burkina Faso might be partly mediated by the closure of health centres. Unfortunately, there is no centralised data in Burkina Faso on the precise dates of closure and reopening of health centres that could confirm this mediation effect.

The impact on service supply is particularly concerning given that demand for contraceptive methods tends to increase during periods of insecurity.^23^ In crisis situations, households often wish to delay pregnancies, while sexual violence and unintended pregnancies tend to rise, increasing the need for contraception and abortion services, as documented in several conflict settings, including in the Sahel.^27 39 86 87^ However, while demand might increase, access to FP services can be hindered, even when they remain available. The presence of armed groups creates a climate of fear that discourages women from visiting health facilities.^88^ Economic barriers, which still exist despite free care policies due to residual costs, are also exacerbated as a result of more severe household financial hardship.^49^ The spread of conservative norms—often promoted by jihadist groups in the Sahel region—may heighten the social stigma surrounding contraceptive services.^89^ Displaced and refugee women are particularly affected by these challenges.^85 90–93^

The combination of increased demand and reduced availability and access to FP services likely worsens UNC in Burkina Faso,^53^ potentially exacerbating the country’s already heavy burden of maternal and infant mortality,^94 95^ as observed in other conflict-affected areas (northern Uganda, Mali and Darfur in Sudan).^27 88 96^

These dual mechanisms—reduced supply and increased access barriers in a context of rising demand—are consistent with Kruk *et al*’s health system resilience framework,^44^ showing how insecurity weakens absorptive and adaptive capacities. In Burkina Faso, the combination of socio-political instability and a large-scale humanitarian crisis^89^ has undermined the health system’s functioning, despite its proven resilience. This situation underscores the need for transformative strategies that integrate FP into broader humanitarian and security-sensitive health system responses grounded in a resilience-oriented approach.

It is important to note that contexts where service supply and access are preserved despite conflict can paradoxically lead to increased contraceptive use and a reduction in unmet need.^39 86 87 97 98^ This suggests that service availability and accessibility play a key intermediary role between need and use. This interaction underscores the importance of a contextualised understanding of the impact of conflict and insecurity on health.

### Passive and active interventions removing user fees

The passive intervention in Burkina Faso (ie, the free FP policy) immediately increased the volume of new FP users by 22% and positively affected the trend increase in the subsequent months. These results are more modest than those measured in a pre-post study, which showed an 86% increase in FP use 8 months after the introduction of the policy.^53^ This difference in timing likely explains the heterogeneous effects: The introduction of the policy was not communicated by mass media, and almost 55% of the target population was still unaware of its existence 6 months after its introduction.^49 53^ It is therefore plausible that the effects of the policy were more gradual than immediate.

Even if the immediate effect was moderate in our study, it was still present and statistically significant: Our results suggest a positive effect of user fee removal on the use of contraception. This conclusion differs from that of a randomised controlled trial conducted between 2018 and 2021, in which no effect was attributed to making contraceptives free of charge.^99^ However, as acknowledged by the authors, the introduction of the national policy in July 2020 has made it impossible to continue to distinguish between the intervention and the control arms in the trial, causing a contamination bias and blurring the measure of effect. Another study,^100^ which focused on the free FP pilot in only two regions over a relatively short period (June 2018–November 2019), was also inconclusive about the policy’s effectiveness.

Taken together, these findings are not contradictory but rather complementary. They suggest that user-fee removal for FP should be regarded as a necessary yet insufficient intervention in protracted crises. Consistent with evidence from humanitarian SRHR programmes, fee exemptions need to be coupled with proactive outreach strategies specifically designed to overcome insecurity-related barriers and to reach women who are most affected by displacement and violence.^101^

To our knowledge, this study is the first to measure the effects of NFPWs (the active intervention) using a quasi-experimental design. We found an immediate 152% increase in the volume of new FP users in the months when an NFPW is held. Interestingly, this increase did not differ between the periods before and after the introduction of the free FP policy (rate ratio=0.99, 95% CI 0.97 to 1.03), suggesting that the two initiatives have additive rather than substitutive effects (online supplemental appendix 3). With its proactive approach to certain groups of women (teenagers, women in remote areas), it is plausible that FP weeks succeed in targeting categories of women that are not reached by the no-cost policy, a passive initiative likely to affect women who live close to a health centre or who are less afraid of being stigmatised. This combination would help avoid what is known as the equity trap, whereby a new health intervention aimed at the entire population is first taken up by better-off groups before slowly reaching the more disadvantaged populations, thereby widening, rather than reducing, health inequalities in the short term.^102 103^

Organisations that have contributed to FP days in Mauritania and elsewhere in Francophone Africa^54^ reported that, in addition to improving access to contraception, these events have budgetary and logistical advantages. For example, as one-off operations, additional security measures can be taken, which is convenient in polycrisis contexts such as Burkina Faso.^104 105^ Because of their effectiveness and positive impact on access inequities, the feasibility of combining NFPWs with other periodic public health activities—such as seasonal malaria chemoprevention or national immunisation days—could be explored. This could further reduce logistical costs and leverage existing community mobilisation infrastructure. However, such integration would need to be approached with caution: it could reinforce community myths linking immunisation campaigns to covert sterilisation, which have been documented in several SSA contexts.^106–108^ Any integration would therefore require careful formative research, clear separation of messages and active engagement of community and religious leaders to prevent undermining trust in either programme.

While user-fee removal contributes to enhancing the services’ affordability and availability, terrorist violence undermines women’s ability to seek and reach care and likely erodes the acceptability of FP services.

### Strengths and limitations

The study is based on a robust quasi-experimental design using long-term national routine data (132 months) and linkage with an established conflict event dataset. Its internal validity was strengthened by using longitudinal regression adjusted for seasonality and fixed effects to control for variables (observed or not) that remained constant at the commune level. However, the study has several limitations. First, it uses secondary data that were collected in a context of insecurity, which may have influenced their quality and completeness. Our sensitivity analyses showed that missing data in the commune increased in the month following an attack. While this is likely due to the temporary closing of health facilities, we could not confirm the hypothesis that the missing data implied a lack of use of services. Data were not available for several months in 2019 when a national healthcare workers’ strike took place; during that time, health services continued to be available and used, but registers were no longer completed. In view of the long observation period under study and the potential bias introduced by any imputation, we decided not to proceed with the replacement of these missing data.

ACLED data also has some limitations: It is difficult to record all attacks accurately and obtain precise information about when and where they occurred.^109 110^ These limitations have become more severe in recent years due to increasing censorship and frequent pressure on the Burkinabe media. The imprecision of the data could be partially overcome by conducting the analyses at a higher spatiotemporal level (eg, the commune-month).

However, the ecological nature of our analyses prevented examination of individual-level determinants, and no conclusions can therefore be drawn about the specific influence of terrorist attacks on women’s contraceptive behaviour or practices. To address this limitation, an in-depth qualitative study is currently underway in Burkina Faso. This qualitative component is explicitly designed to complement the quantitative analyses by unpacking the micro-level mechanisms underlying the observed population-level associations, thereby strengthening the interpretation of our findings.

This study measured only the average immediate effect of the occurrence of attacks; repeated attacks in some communes could plausibly generate heterogeneous effects—that is, effect sizes that vary across communes according to the frequency or intensity of attacks, the presence of internally displaced populations or distance to the nearest functioning health facility. Finally, it was not possible to consider the variation in the catchment population at the commune level. Population shifts could explain the lower volume of consultations in insecure areas. However, our investigations at higher levels of aggregation indicate that attacks affect the total volume of new FP users, suggesting a net reduction rather than perfect substitution between communes.

## Conclusion

While free FP services can increase uptake of contraceptive methods, terrorist attacks and insecurity cause major disruptions on both the supply and demand sides, thereby undermining their use. User-fee removal should therefore be embedded within broader strategies to protect and adapt FP services in conflict-affected areas, including securing facilities, supporting community health workers as local relays, promoting self-care where appropriate and integrating FP into humanitarian and outreach campaigns.

Beyond Burkina Faso, insecurity has become a structural determinant of health across the Sahel and can no longer be treated as a peripheral constraint. Achieving the FP2030 agenda in this region will require explicitly tailoring FP policies and programmes to contexts of chronic vulnerability. In this regard, the demonstrated effectiveness of intensive, time-limited interventions such as NFPWs suggests that combining innovative delivery strategies with user-fee removal constitutes a critical lever for sustaining contraceptive access.

FP policy and NFPWs have generated substantial gains in new contraceptive users at the national level. In the current insecurity context, reducing financial or programmatic support for FP is therefore likely to exacerbate UNC and to undermine progress in maternal and child health, particularly among the most vulnerable women living in insecure or remote areas.

## Supplementary material

10.1136/bmjopen-2026-116769 online supplemental file 1

10.1136/bmjopen-2026-116769 online supplemental file 2

10.1136/bmjopen-2026-116769 online supplemental file 3

10.1136/bmjopen-2026-116769 online supplemental file 4

## Data Availability

Some data are available in a public, open access repository. Some data may be obtained from a third party and are not publicly available.
