# Supplementary material for: Effects of terrorism-related attacks and family planning interventions on the uptake of modern contraceptives in Burkina Faso: a nationwide interrupted time-series analysis (2013–2023)
Source: BMJ Open. 2026 Aug 31;16(8):e116769. doi: 10.1136/bmjopen-2026-116769 (PMC13536019; doi:10.1136/bmjopen-2026-116769)
Supplement: Supplementary file 1 [file bmjopen-2026-116769.reviewer_comments.pdf]

PEER REVIEW HISTORY

BMJ Open publishes all reviews undertaken for accepted manuscripts. Reviewers are asked to complete a checklist review form and are provided with free text boxes to elaborate on their assessment. These free text comments are reproduced below.

This paper was submitted to another journal from BMJ but declined for publication following peer review. The authors addressed the reviewers' comments and submitted the revised paper to BMJ Open. The paper was subsequently accepted for publication at BMJ Open.

ARTICLE DETAILS

Title (Provisional)

Effects of terrorism-related attacks and family planning interventions on the uptake of modern contraceptives in Burkina Faso: A nationwide interrupted time-series analysis (2013–2023)

Authors

Tiendrebeogo, Cheick; Fregonese, Federica; Firouraghi, Neda; Druetz, Thomas

---

VERSION 1 - REVIEW

**Reviewer**	**1**
**Name**	**Reviewer 1**
**Affiliation**	
**Date**	**15-Dec-2025**
**COI**	

Comments not published pending reviewer's consent

**Reviewer**	**2**
**Name**	**Reviewer 2**
**Affiliation**	
**Date**	**15-Dec-2025**
**COI**	

Comments not published pending reviewer's consent

VERSION 2 - REVIEW

**Reviewer**	**3**
**Name**	**Bertrand, Jane**
**Affiliation**	**Tulane University**
**Date**	**25-Mar-2026**
**COI**	**None**

This article explores an important and novel topic: The impact of terrorist attacks on the use of modern contraceptive methods and the mitigating effects of recent public health interventions in Burkina Faso. It uses robust data sets (the DHIS2, Armed Conflict Location and Event Data, and the Humanitarian Data Exchange Platform, among others). This longitudinal study employs multiple interrupted time series, which is appropriate to the research questions. It measures the relative impact of terrorist attacks, National FP weeks, and removal of user fees on contraceptive uptake, which is innovative and important. The paper is well organized and well-written. The discussion is comprehensive and thoughtful. And the list of references is extensive and appropriate.

The major limitation of the paper is the dependent variable: new users. This indicator has been widely maligned in the family planning literature, since it has alternative definitions/interpretations, and service providers do not necessarily use it consistently. More important, it does not capture the volume of service being delivered because the ratio of new users to all users is not consistent over time or by geography.

A related issue is the mention of “all users” on line 263 (without defining it in the methodology). This indicator sounds intuitive and plausible, but “all users” cannot be reliably measured from the DHIS2 or other service statistics data files. Does the Burkina Faso DHIS2 contain data that allow for the measurement of couple-years of protection? It would be the far more appropriate measure for this analysis. Even the number of FP visits would be preferable to new users.

Other observations (note: the page numbers correspond to the pdf, not to the original manuscript):

Lines 393-396: The authors suggest combining family planning events with seasonal malaria or national immunization days. However, such a combination may trigger myths common in some countries that immunizations contain ingredients that sterilize the population.

In several places, the terms used may be beyond the understanding of some readers; the authors should explain in lay terms:

- Line 139: commun-month level
- Line 387: reverse equity
- Line 395: heterogeneous effects

The paper was refreshingly innovative, but the authors should be required to reconsider the dependent variable before publication.

**Reviewer**	**4**
**Name**	**Kelson, Zoe**
**Affiliation**	**University of Exeter, Mathematics**
**Date**	**21-May-2026**
**COI**	**None**

This study investigates the impact of terrorist attacks on the use of modern contraceptive methods and the mitigating effects of recent public interventions in Burkina Faso.

Reviewer comments:

"This study has followed the RECORD (REporting of studies Conducted using Observational Routinely-collected health Data) guidelines"

Thanks to the authors for providing a copy of the STROBE checklist.

"Monthly data for all public primary healthcare centers and district hospitals on new family planning (FP) users were drawn from Burkina Faso's National Health Information System, covering 132 months (January 2013–December 2023)" [Abstract]

and

"Health services data from January 2013 to December 2023 (132 months) were collated at the commune level (the smallest administrative unit in the country)... Data on contraceptive users were obtained from Burkina Faso's national health information system (NHIS) database."

The completeness and coverage of the data has been addressed by the authors.

"These consultation data are collected monthly at all public health centers in Burkina Faso: ~2300 primary healthcare facilities, 49 district-level medical centers, nine regional hospitals, and six university hospitals. "

Can clustering in the data (i.e. by facility, center, hospital and university hospital) please be suitably adjusted for in the analysis (e.g. applying multilevel modlleing)?

"a longitudinal study using a multiple interrupted time series analysis... A negative binomial regression model with commune-level fixed effects, seasonality adjustment, and autocorrelation correction was fitted to test the effects of attacks, user-fee removal, and National Family Planning Weeks (NFPWs) on the monthly count of new FP users." [Abstract]

and

"This is a longitudinal study using multiple interrupted time series performed on routine administrative data collected monthly in all public health centers in Burkina Faso...

Interrupted time series were fitted using a longitudinal panel regression framework with commune and month fixed effects (to account for unobserved heterogeneity and seasonality), within a negative binomial specification to address over-dispersion. A baseline trend parameter was added in the model, and robust variance estimators were applied throughout. The model also incorporates two dummy variables to assess (i) the immediate effect of introducing the free FP policy and (ii) the change in the baseline trend after its introduction. Finally, a dummy variable was added to measure the average treatment effect of being exposed to NFPWs. Quadratic terms were tested to allow a curvilinear function of the baseline and post-gratuity trends. Residual serial correlation was reduced by using fixed effects and adding a first-order autocorrelation parameter. Coefficients were expressed as incidence rate ratios. The models derive from...

Sensitivity analyses were performed to test the hypothesis that attacks can lead to the temporary closure of health centers, causing a reduction in the rate of FP consultations. A regression model was fitted using a Poisson distribution with the dependent variable being the number of health facilities per commune that transmitted consultation data. Fixed effects were included at the commune level. "

The modelling approach applied is clearly described in detail by the authors.

"Table 1 Effects of attacks and interventions on new FP users in Burkina Faso, 2013- 242 2023"

and

"The rate of new FP users in a commune fell by an average of 13% (95% CI 11%–15%) during the months when a terrorist attack was recorded. The sensitive analysis shows that attacks were also associated with a reduction in the number of health facilities reporting data (-15%, 95% CI 14%–17%) (Appendix 2). "

and

"Table 2 Predicted gains and losses in the total number of new FP users in Burkina 285 Faso, 2013-2023"

and

"Appendix 3 Results of Interaction between free FP and NFPWs (2013-2023)"

Uncertainty has been suitably quantified and communicated by the authors.

"Figure 1 Total number of users (new & old) of family planning services in Burkina Faso, per year (2013-2023)"

Can uncertainty (e.g. confidence intervals) please be shown on this plot?

"A visual examination of the crude trend in the total number of new FP users in Burkina Faso shows a significant increase each month 252 an NFPW was held (Figure 3)"

Can the authors please refrain from inferring a 'significant increase' for descriptive analyses (can this please be checked throughout the Results)?

"Strengths and Limitations "

The authors provide a discussion on the study limitations.

VERSION 2 - AUTHOR RESPONSE

Reviewer 3 — Dr. Jane Bertrand (Tulane University)

We thank Dr. Bertrand for her detailed and very constructive review, particularly her recognition of the originality of the research question and the appropriateness of the design, and the critical reflection on the choice of dependent variable, which has been central to our revision.

2.1 Major comment — Choice of dependent variable (“new users”)

> *The major limitation of the paper is the dependent variable: “new users”. This indicator has been widely maligned in the family planning literature, since it has alternative definitions/interpretations, and service providers do not necessarily use it consistently. More important, it does not capture the volume of service being delivered because the ratio of new users to all users is not consistent over time or by geography. A related issue is the mention of “all users” on line 263 (without defining it in the methodology). This indicator sounds intuitive and plausible, but “all users” cannot be reliably measured from the DHIS2 or other service statistics data files. Does the Burkina Faso DHIS2 contain data that allow for the measurement of couple-years of protection? It would be the far more appropriate measure for this analysis. Even the number of FP visits would be preferable to new users.*

Response. We thank the reviewer for this important comment. We acknowledge that the definitions of the variables "new users" and "all users" were not sufficiently clear in the original manuscript.

In the Burkina Faso National Health Information System (NHIS), the indicator **“new FP users”** represents the number of visits for family planning services by women recorded for the first time as adopting a modern contraceptive method in a given health facility. The indicator **“total FP users”** (formerly referred to as “all users”) represents the total number of family planning service visits, including both new users and old/continuing users returning for follow-up, resupply, or continuation of a modern contraceptive method.

Because our objective was to assess the uptake of modern contraception and its association with insecurity and family planning policies, we retained **new FP users** as the primary outcome. We have clarified the definitions of both indicators in the Methods section and replaced the ambiguous term “all users” throughout the manuscript.

We also retained Appendix 4 as a descriptive comparison of annual trends in new FP users and total FP users (new plus old/continuing users). This appendix provides contextual information on overall family planning service utilisation.

We agree that other indicators, such as couple-years of protection (CYP), may provide complementary information on the volume of contraceptive services delivered. However, these data were not available. This limitation is now acknowledged in the Discussion.

Changes in manuscript: Methods \ Data Sources and Outcome Variables (page 9); Discussion (page 16).

2.2 Combining NFPWs with malaria chemoprophylaxis or immunisation days

> *The authors suggest combining family planning events with seasonal malaria or national immunization days. However, such a combination may trigger myths common in some countries that immunizations contain ingredients that sterilize the population.*

Response. This is an important caveat. We have substantially revised the corresponding paragraph to acknowledge this risk and to soften the recommendation. The revised text reads:

> *"Because of their effectiveness and positive impact on access inequities, the feasibility of combining NFPWs with other periodic public health activities — such as seasonal malaria chemoprevention or national immunisation days — could be explored. This could further reduce logistical costs and leverage existing community mobilisation infrastructure. However, such integration would need to be approached with caution: it could reinforce community myths linking immunisation campaigns to covert sterilisation, which have been documented in several sub-Saharan African contexts (Muanda et al. 2016 and 2017; Renne 2010). Any integration would therefore require careful formative research, clear separation of messaging and active engagement of community and religious leaders to prevent the erosion of trust in either programme."*

Change in manuscript: Discussion — *Passive and active interventions* (page 19)

2.3 Lay-language explanations

> *In several places, the terms used may be beyond the understanding of some readers; the authors should explain in lay terms: • Line 139: commune-month level • Line 387: reverse equity • Line 395: heterogeneous effects*

Response. We thank the reviewer for this helpful suggestion. We have inserted plain-language explanations at the first mention of each of these terms.

(a) Commune-month level (Methods).

> *"Throughout this study, the unit of analysis is the* ***commune-month*** *— that is, observations per commune (the smallest administrative subdivision of Burkina Faso; n = 351) for each calendar month over 132 months. Each commune-month contains the count of new family planning users aggregated across all public health facilities located in that commune."*

(b) Reverse equity / inverse equity hypothesis (Discussion, page, line).

> *"This combination would help avoid what is known as the* ***'inverse equity' or 'reverse equity' trap,*** *whereby a new health intervention aimed at the entire population is first taken up by better-off groups before slowly reaching the more disadvantaged, thereby widening, rather than reducing, health inequalities in the short term."*

(c) Heterogeneous effects (Discussion — Limitations, page, line).

> *"Repetition of attacks in certain communes could plausibly lead to different patterns of insecurity and trigger* ***effect sizes that vary across subgroups of communes (for example, according to the frequency or intensity of attacks, the presence of internally displaced populations, or distance to the nearest functioning health facility)****. This question is the focus of an ongoing follow-up analysis."*

Change in manuscript: Methods; Discussion.

Reviewer 4 — Prof. Zoe Kelson (University of Exeter)

We thank Prof. Kelson for her positive assessment of the data, design and modelling, and for her constructive methodological and reporting suggestions.

3.1 Clustering and multilevel modelling

> *Can clustering in the data (i.e. by facility, center, hospital and university hospital) please be suitably adjusted for in the analysis (e.g. applying multilevel modelling)?*

Response. We thank the reviewer for this suggestion. However, the objective of this study was to examine family planning service utilisation at the **commune-month level**, not at the health-facility level. The analytical dataset was therefore aggregated at the commune level, and therefore clustering at the facility-level can no longer be adjusted for. Multi-level models with fixed effects at the commune-level were used instead, with cluster-robust variance estimators. This clarification has been added to the Methods section.

Changes in manuscript: Methods – Analysis (page, line).

3.2 Uncertainty / confidence intervals on the figure of total FP users

> *Figure 1 Total number of users (new & old) of family planning services in Burkina Faso, per year (2013-2023). Can uncertainty (e.g. confidence intervals) please be shown on this plot?*

Response. We thank the reviewer for this suggestion. We did not add confidence intervals to the Appendix 4 - Figure 1, as it does not present estimates but rather the total counts extracted from the NHIS database, adding the visits of each month per year. The caption now explicitly states that it presents "raw annual aggregates extracted from the NHIS", and we have removed any inferential language from the figure caption and from the corresponding paragraph in the Results (see also point 3.3 below).

Change in manuscript: descriptive yearly figure moved to Appendix 4 — Figure 1 and captions revised (pages, lines).

3.3 "Significant" used in descriptive analyses

> *A visual examination of the crude trend in the total number of new FP users in Burkina Faso shows a significant increase each month an NFPW was held. (…) Can the authors please refrain from inferring a 'significant increase' for descriptive analyses (can this please be checked throughout the Results)?*

Response. We agree with the comment and have removed the word "significant" (and its variants) from every sentence referring to descriptive observations, throughout the Results and figure captions, reserving the term exclusively for findings emerging from formal statistical tests.

In addition to the sentence identified, edits were done to four sentences in Results and to the captions of Figure 3 and Appendix 4 — Figure 1.

Change in manuscript: Results, throughout; Figure 3 and Appendix 4 — Figure 1 captions. (pages, lines)

We thank the Editor and the Reviewers once again for their careful and constructive engagement with our work. We remain at your entire disposal for any further clarification.

VERSION 3 - REVIEW

**Reviewer**	**3**
**Name**	**Bertrand, Jane**
**Affiliation**	**Tulane University**
**Date**	**06-Jul-2026**
**COI**	

Reviewer comments: Burkina Faso article

The authors have addressed most of my comments from the previous review. However, they need to address two problems with the FP indicators.

Allow me to continue to nitpick about the dependent variable. Lines 184–187, the authors clearly define a "new user" as "any woman recorded for the first time as adopting a modern contraceptive method at a given health facility." And they define the primary outcome as the monthly number of visits for new FP users per commune-month. In the case of new users, one new user equals one visit by a new user, so there is no problem. The same logic does not hold for all FP users, because a given continuing user could return four times a year to receive an injectable, which would be equivalent to four visits, but only one continuing user.

My guess is that the Burkina Faso DHIS2 conflates total number of visits for all FP users with total number of FP users. If this is the case, the authors have a bit of a dilemma. Do they use the terms as defined in the Burkina Faso DHIS2, or do they try to be as accurate as possible by replacing "all FP users" with "the total number of FP visits by all users" (or simply "user visits").

Lines 310–313: This result would be more acceptable if the authors referred to the total number of family planning visits rather than the total number of FP users.

One way to handle this problem is the use "new FP user" as the primary dependent variable but change to total FP visits when presenting the latter. A second alternative (which in my opinion would be preferable) would be to use "number of visits" as the outcome variable for both first time users and all users.

This is a minor problem within an excellent article, which will be a great contribution to the family planning literature. However, it still contains this one problem of equating total number of users with total number of visits by FP users.

Line 280 -282: "the rate of new FP users" - A rate requires both numerator and a denominator, whereas this analysis uses the number of service visits by new FP users. If there is a denominator, it's unclear what it is.

Line 304: Again, what is the rate of a new user? Isn't it an increase in the number of new user visits?

Otherwise, I have no further comments on this article.

VERSION 3 - AUTHOR RESPONSE

Reviewer 3
The authors have addressed most of my comments from the previous review. However, they need to address two problems with the FP indicators.

Response: We thank Dr Bertrand for her careful second review. We appreciate the additional clarification regarding the distinction between users and visits in routine family planning indicators.

Allow me to continue to nitpick about the dependent variable. Lines 184–187, the authors clearly define a "new user" as "any woman recorded for the first time as adopting a modern contraceptive method at a given health facility." And they define the primary outcome as the monthly number of visits for new FP users per commune-month. In the case of new users, one new user equals one visit by a new user, so there is no problem. The same logic does not hold for all FP users, because a given continuing user could return four times a year to receive an injectable, which would be equivalent to four visits, but only one continuing user. My guess is that the Burkina Faso DHIS2 conflates total number of visits for all FP users with total number of FP users. If this is the case, the authors have a bit of a dilemma. Do they use the terms as defined in the Burkina Faso DHIS2, or do they try to be as accurate as possible by replacing "all FP users" with "the total number of FP visits by all users" (or

simply “user visits”). Lines 310–313: This result would be more acceptable if the authors referred to the total number of family planning visits rather than the total number of FP users. One way to handle this problem is the use “new FP user” as the primary dependent variable but change to total FP visits when presenting the latter. A second alternative (which in my opinion would be preferable) would be to use “number of visits” as the outcome variable for both first time users and all users. This is a minor problem within an excellent article, which will be a great contribution to the family planning literature. However, it still contains this one problem of equating total number of users with total number of visits by FP users.

Response: We thank Dr Bertrand for this important clarification. We have revised the manuscript to avoid equating total users with total visits. Throughout the manuscript and Appendix 4, we now refer to this indicator as “total FP visits” or “visits by new and old/continuing FP users”, rather than “all FP users” or “total FP users.”
Change in manuscript: lines 53 and 321-324 (previously 310-313), and Appendix 4.

Line 280 -282: “the rate of new FP users” - A rate requires both numerator and a denominator, whereas this analysis uses the number of service visits by new FP users. If there is a denominator, it’s unclear what it is. Line 304: Again, what is the rate of a new user? Isn’t it an increase in the number of new user visits?

Response: We thank Dr Bertrand and agree with her suggestion. To avoid ambiguity, we have removed the expression “rate of new FP users”. We now refer to the “monthly number of visits by new FP users”.
Change in manuscript: lines 27, 290-291 (previously 280-282), and 315 (previously 304).
