# Supplementary material for: Effects of terrorism-related attacks and family planning interventions on the uptake of modern contraceptives in Burkina Faso: a nationwide interrupted time-series analysis (2013–2023)
Source: BMJ Open. 2026 Aug 31;16(8):e116769. doi: 10.1136/bmjopen-2026-116769 (PMC13536019; doi:10.1136/bmjopen-2026-116769)
Supplement: Supplementary file 2 [file bmjopen-16-8-s001.docx]

Appendix 1 (Supplementary figure 1) RECORD flow diagram of data linkage and analytical sample selection

This diagram summarises the routine health information data, conflict-event data and contextual sources used to construct the final commune-month analytical panel for the multiple interrupted time-series analysis.

**1. National Health Information System (NHIS), Burkina Faso**

• Period: January 2013-December 2023

• Source population: all public health facilities in Burkina Faso (~2300 primary healthcare facilities, 49 district-level medical centres, 9 regional hospitals and 6 university hospitals)

• Variables extracted: monthly number of visits of new FP users, monthly number of visits of old/continuing FP users, and monthly number of facilities reporting consultation data

• Processing: facility-level monthly data* on volume of visits aggregated to the commune-month level

• Data were routinely collected for the NHIS

**↓**

**2. Armed Conflict Location and Event Data (ACLED)**

• Period: January 2013-December 2023

• Events screened: all events recorded in ACLED in Burkina Faso

• Excluded: riots, protests and strategic developments.

• Retained: battles, explosions/remote violence and violence against civilians

• Processing: geocoded events assigned to communes and calendar months

**↓**

**3. Additional contextual sources**

• National Family Planning Weeks (NFPWs): campaign dates compiled from Ministry of Health and UNFPA sources

• Population denominators: 2019 General Population and Housing Census of Burkina Faso

• Administrative boundaries and commune correspondence: HDX/OCHA boundaries cross-checked with WHO and national health information sources

**↓**

**4. Harmonisation and linkage**

• Commune names/codes harmonised across NHIS, ACLED and census-related sources

• ACLED events linked to NHIS observations by geography (commune) and time (calendar month)

• NFPW exposure and free FP policy variables added at the commune-month level

• Final unit of analysis: commune-month

**↓**

**5. Final analytical panel**

• 351 communes with data for 132 months

• Maximum analytical structure: 46,332 commune-month observations

• Terrorist attacks recorded in 254/351 communes and in 117/132 monthly observation periods

• NHIS data missing during the 2019 nationwide healthcare workers' strike were retained as missing and not imputed

**↓**

**6. Analyses reported in the manuscript and supplementary material**

• Main analysis: multiple interrupted time-series negative binomial regression with commune fixed effects, month fixed effects, seasonality adjustment and autocorrelation correction

• Primary outcome: monthly number of visits for new FP users

• Appendix 2: sensitivity analysis on health-facility data transmission

• Appendix 3: interaction between free FP policy and NFPWs

• Appendix 4: descriptive annual totals of visits for new and returning FP users

FP, family planning; NHIS, National Health Information System; ACLED, Armed Conflict Location and Event Data; NFPWs, National Family Planning Weeks; HDX, Humanitarian Data Exchange; OCHA, United Nations Office for the Coordination of Humanitarian Affairs; UNFPA, United Nations Population Fund.
