# Supplementary material for: Effects of terrorism-related attacks and family planning interventions on the uptake of modern contraceptives in Burkina Faso: a nationwide interrupted time-series analysis (2013–2023)
Source: BMJ Open. 2026 Aug 31;16(8):e116769. doi: 10.1136/bmjopen-2026-116769 (PMC13536019; doi:10.1136/bmjopen-2026-116769)
Supplement: Supplementary file 4 [file bmjopen-16-8-s003.docx]

**Appendix 3 Results of Interaction between free FP and NFPWs (2013-2023)**

| Visits of new FP users | IRR | 95% CI |
| --- | --- | --- |
| Secular trend | 0.990*** | 0.989 0.990 |
| Secular trend (quadratic) | 1.000*** | 1.000 1.000 |
| Free FP (yes) | 1.215*** | 1.183 1.248 |
| NFPW (yes) | 2.516*** | 2.459 2.576 |
| Interaction Free FP (yes) & NFPW (yes) | 0.998 | 0.970 1.028 |
| Post-gratuity trend | 1.007*** | 1.005 1.009 |
| Post-gratuity trend (quadratic) | 0.999*** | 0.999 0.999 |
| Terrorist attacks (yes) | 0.872*** | 0.856 0.890 |

***p<0.001; IRR incidence rate ratio; CI Confidence interval. Results obtained by using negative binomial regression model with robust variance estimators and fixed effects at the commune level and adjusted for seasonal variations (months). The exposure variable is categorical and expressed by interaction between free FP and national FP weeks.
