# Supplementary material for: Effects of terrorism-related attacks and family planning interventions on the uptake of modern contraceptives in Burkina Faso: a nationwide interrupted time-series analysis (2013–2023)
Source: BMJ Open. 2026 Aug 31;16(8):e116769. doi: 10.1136/bmjopen-2026-116769 (PMC13536019; doi:10.1136/bmjopen-2026-116769)
Supplement: Supplementary file 5 [file bmjopen-16-8-s004.docx]

Appendix 4 (Table 1) Total number of family planning users visits in Burkina Faso, per year (2013-2023)

| Year | Visits by old/continuing PF users | Visits by new PF users | Total FP users visits |
| --- | --- | --- | --- |
| 2013 | 630,576 | 400,332 | 1,030,908 |
| 2014 | 666,723 | 440,717 | 1,107,440 |
| 2015 | 701,738 | 357,281 | 1,059,019 |
| 2016 | 796,741 | 369,385 | 1,166,126 |
| 2017 | 682,770 | 349,730 | 1,032,500 |
| 2018 | 624,862 | 343,016 | 967,878 |
| 2019* | 343,490* | 167,897* | 511,387 |
| 2020 | 657,021 | 278,710 | 935,731 |
| 2021 | 759,408 | 339,459 | 1,098,867 |
| 2022 | 671,764 | 320,264 | 992,028 |
| 2023 | 657,757 | 316,811 | 974,568 |
| Total | 7,192,850 | 3,683,602 | 10,876,452 |

FP Family planning; * In 2019, a general strike by health workers affected data collection for 5 months, therefore the data reported should be considered only for 7 months in 2019

Appendix 4 (Figure 1) Trends in family planning users visits (new, old/continuing, total) in Burkina Faso, per year (2013-2023)
